# Chloridobis(2,9-dieth­oxy-1,10-phenanthroline-κ^2^ *N*,*N*′)copper(II) perchlorate

**DOI:** 10.1107/S160053680804138X

**Published:** 2008-12-13

**Authors:** Xin-Sheng Wan, Lei Meng, Chao-Ling Feng, Chun-Hong Kou, Cao-Yuan Niu

**Affiliations:** a College of Sciences, Henan Agricultural University, Zhengzhou 450002, People’s Republic of China

## Abstract

In the title complex, [CuCl(C_16_H_16_N_2_O_2_)_2_]ClO_4_, the Cu^II^ ion is coordinated by four N atoms from two chelating 2,9-dieth­oxy-1,10-phenanthroline ligands and one chloride ion in a slightly disorted trigonal-bipyramidal environment. Two N atoms and the Cl atom are in equatorial positions while the remaining two N atoms occupy apical sites, the equatorial Cu—N bonds being significantly longer than the two apical Cu—N bonds. The N=C—O—C torsion angles involving the four eth­oxy groups are in the range 161.5 (8) to 177.0 (5)°. In the crystal structure, there are significant π–π stacking inter­actions between inversion-related rings of phenanthroline groups with centroid–centroid distances in the range 3.649 (4)–3.790 (4) Å.

## Related literature

For background information, see: Pijper *et al.* (1984 ▶).
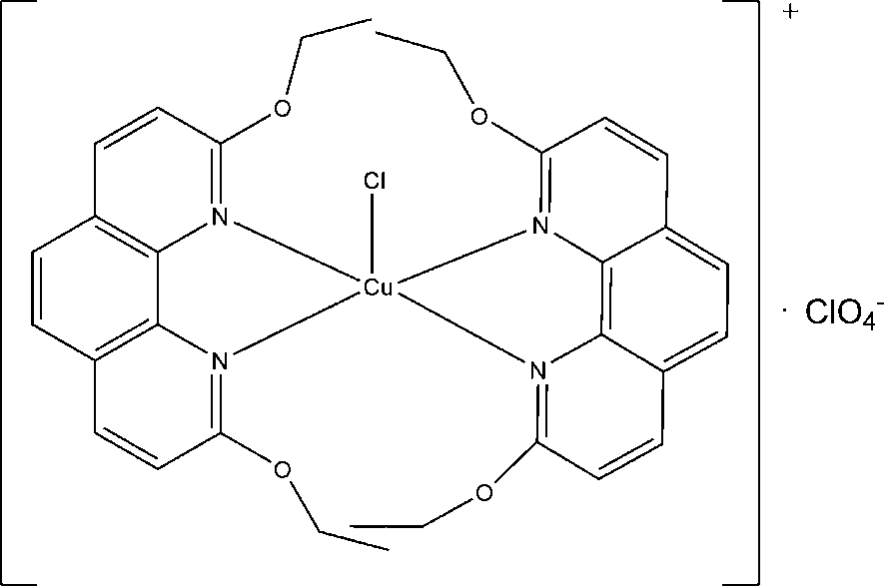


## Experimental

### 

#### Crystal data

[CuCl(C_16_H_16_N_2_O_2_)_2_]ClO_4_
*M* *_r_* = 735.06
Monoclinic, 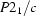

*a* = 9.7461 (13) Å
*b* = 23.953 (3) Å
*c* = 13.9777 (18) Å
β = 91.837 (2)°
*V* = 3261.4 (7) Å^3^
*Z* = 4
Mo *K*α radiation
μ = 0.89 mm^−1^
*T* = 291 (2) K
0.34 × 0.27 × 0.17 mm

#### Data collection

Siemens SMART CCD area-detector diffractometer
Absorption correction: multi-scan (*SADABS*; Sheldrick, 1996 ▶) *T*_min_ = 0.750, *T*_max_ = 0.863
16840 measured reflections
6045 independent reflections
3209 reflections with *I* > 2σ(*I*)
*R*_int_ = 0.055

#### Refinement

*R*[*F* ^2^ > 2σ(*F* ^2^)] = 0.065
*wR*(*F* ^2^) = 0.216
*S* = 1.05
6045 reflections
428 parameters
100 restraints
H-atom parameters constrained
Δρ_max_ = 0.67 e Å^−3^
Δρ_min_ = −0.43 e Å^−3^

### 

Data collection: *SMART* (Siemens, 1996 ▶); cell refinement: *SAINT* (Siemens, 1994 ▶); data reduction: *SAINT*; program(s) used to solve structure: *SHELXS97* (Sheldrick, 2008 ▶); program(s) used to refine structure: *SHELXL97* (Sheldrick, 2008 ▶); molecular graphics: *PLATON* (Spek, 2003 ▶) and *DIAMOND* (Brandenburg, 2005 ▶); software used to prepare material for publication: *SHELXTL* (Sheldrick, 2008 ▶).

## Supplementary Material

Crystal structure: contains datablocks I, global. DOI: 10.1107/S160053680804138X/lh2739sup1.cif

Structure factors: contains datablocks I. DOI: 10.1107/S160053680804138X/lh2739Isup2.hkl

Additional supplementary materials:  crystallographic information; 3D view; checkCIF report

## Figures and Tables

**Table:** 

Cu1—N1	1.999 (5)
Cu1—N4	2.003 (5)
Cu1—N2	2.158 (5)
Cu1—N3	2.162 (5)
Cu1—Cl1	2.2847 (19)

**Table:** 

N1—Cu1—N4	176.8 (2)
N1—Cu1—N2	80.38 (19)
N4—Cu1—N2	102.79 (19)
N1—Cu1—N3	100.03 (19)
N4—Cu1—N3	79.6 (2)
N2—Cu1—N3	101.22 (17)
N1—Cu1—Cl1	91.64 (15)
N4—Cu1—Cl1	86.22 (15)
N2—Cu1—Cl1	130.20 (13)
N3—Cu1—Cl1	128.54 (15)

**Fig. 1.**
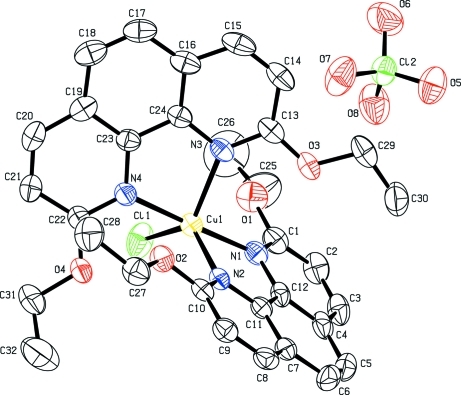
The molecular structure of the title complex, showing the atom-labelling scheme. Displacement ellipsoids are drawn at the 30% probability level and H atoms are not shown.

**Fig. 2.**
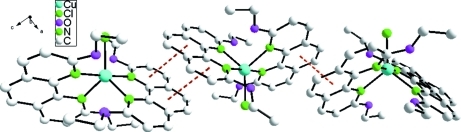
Part of the crystal structure showing intermolecular π···π stacking indicated by dashed lines. All H atoms and perchlorate anions have been omitted for clarity.

## supplementary crystallographic information

### Comment

The synthesis of 2,9-Dimethoxy-1,10-phenanthroline and 2,9-diethoxy-1,10-phenanthroline have already been reported in the literature and have been shown to possess antimycoplasmal activity in the presence of copper (Pijper, *et al.*, 1984). However, no crystal structures of their copper complexes have so far been reported. Herein we report the crystal structure of a mononuclear copper complex with 2,9-diethoxy-1,10-phenanthroline.

In the title compound, the Cu^II^ ion is coordinated by four nitrogen atoms from two phenanthroline rings (N1, N2, N3, N4) and one chloride ion (Cl1) forming a slightly distorted trigonal-bipyramidal geometry (Fig.1). Atoms N2, N3, and Cl1 are located in the equatorial positions and atoms N1 and N4 in the apical sites. The Cu1—N2 and Cu1—N3 bonds are significantly longer than the other two Cu—N bonds. The N=C—O—C torsion angles involving the four ethoxy groups are in the range 161.5 (8) to 177.0 (5)°. In the crystal structure, significant π···π stacking interactions between pairs of inversion related parallel rings of the phenanthroline groups give centroid-to-centroid distances in the range 3.649 (4)-3.790 (4) Å (Fig. 2). One perchlorate anion acts as the counteranion balancing the charge on the mononuclear complex.

### Experimental

2,9-diethoxy-1,10-phenanthroline was prepared according to the literature procedure (Pijper, *et al.*, 1984). The slow evaporation of a mixture of the ligand (0.024 g, 0.1 mmol), CuCl_2_ (0.016 g, 0.1 mmol), and NaClO_4_^.^6H_2_O (0.037 g, 0.1 mmol) in 30 ml methanol afforded green block single crystals in about 15 days (yield about 70%).

### Refinement

The H atoms were positioned geometrically and refined using a riding model [C—H = 0.93 Å and *U*_iso_(H) = 1.2*U*_eq_(C) for aromatic H atoms; C—H = 0.97 Å and *U*_iso_(H) = 1.2*U*_eq_(C) for methylene H atoms; C—H = 0.96 Å and *U*_iso_(H) = 1.5*U*_eq_(C) for methyl H atoms]. The C atoms of the ethoxy groups have larger displacement parameters than normal. This may be due to mimimal disorder which was not modelled. However, the C—C bond distances in the four methylene groups were constrained to 1.53 (1) Å.

### Crystal data

**Table:**

[CuCl(C_16_H_16_N_2_O_2_)_2_]ClO_4_	*F*(000) = 1516
*M**_r_* = 735.06	*D*_x_ = 1.497 Mg m^−^^3^
Monoclinic, *P*2_1_/*c*	Mo *K*α radiation, λ = 0.71073 Å
Hall symbol: -P 2ybc	Cell parameters from 2218 reflections
*a* = 9.7461 (13) Å	θ = 2.7–25.5°
*b* = 23.953 (3) Å	µ = 0.89 mm^−^^1^
*c* = 13.9777 (18) Å	*T* = 291 K
β = 91.837 (2)°	Block, green
*V* = 3261.4 (7) Å^3^	0.34 × 0.27 × 0.17 mm
*Z* = 4	

### Data collection

**Table:**

Siemens SMART CCD area-detector diffractometer	6045 independent reflections
Radiation source: fine-focus sealed tube	3209 reflections with *I* > 2σ(*I*)
graphite	*R*_int_ = 0.055
φ and ω scans	θ_max_ = 25.5°, θ_min_ = 2.7°
Absorption correction: multi-scan (*SADABS*; Sheldrick, 1996)	*h* = −11→11
*T*_min_ = 0.750, *T*_max_ = 0.863	*k* = −28→29
16840 measured reflections	*l* = −14→16

### Refinement

**Table:**

Refinement on *F*^2^	Primary atom site location: structure-invariant direct methods
Least-squares matrix: full	Secondary atom site location: difference Fourier map
*R*[*F*^2^ > 2σ(*F*^2^)] = 0.065	Hydrogen site location: inferred from neighbouring sites
*wR*(*F*^2^) = 0.216	H-atom parameters constrained
*S* = 1.05	*w* = 1/[σ^2^(*F*_o_^2^) + (0.1064*P*)^2^ + 1.0151*P*] where *P* = (*F*_o_^2^ + 2*F*_c_^2^)/3
6045 reflections	(Δ/σ)_max_ < 0.001
428 parameters	Δρ_max_ = 0.67 e Å^−^^3^
100 restraints	Δρ_min_ = −0.43 e Å^−^^3^

### Special details

**Table:**

Geometry. All e.s.d.'s (except the e.s.d. in the dihedral angle between two l.s. planes) are estimated using the full covariance matrix. The cell e.s.d.'s are taken into account individually in the estimation of e.s.d.'s in distances, angles and torsion angles; correlations between e.s.d.'s in cell parameters are only used when they are defined by crystal symmetry. An approximate (isotropic) treatment of cell e.s.d.'s is used for estimating e.s.d.'s involving l.s. planes.
Refinement. Refinement of *F*^2^ against ALL reflections. The weighted *R*-factor *wR* and goodness of fit *S* are based on *F*^2^, conventional *R*-factors *R* are based on *F*, with *F* set to zero for negative *F*^2^. The threshold expression of *F*^2^ > σ(*F*^2^) is used only for calculating *R*-factors(gt) *etc*. and is not relevant to the choice of reflections for refinement. *R*-factors based on *F*^2^ are statistically about twice as large as those based on *F*, and *R*- factors based on ALL data will be even larger.

### Fractional atomic coordinates and isotropic or equivalent isotropic displacement parameters (Å^2^)

**Table:**

	*x*	*y*	*z*	*U*_iso_*/*U*_eq_	
Cu1	0.26542 (7)	0.47318 (3)	0.74564 (5)	0.0571 (3)	
Cl1	0.07239 (19)	0.47574 (9)	0.64858 (15)	0.0997 (7)	
Cl2	0.26726 (19)	0.73954 (7)	0.91201 (14)	0.0747 (5)	
O1	0.1727 (6)	0.59575 (19)	0.7034 (4)	0.0889 (15)	
O2	0.4526 (4)	0.35968 (16)	0.8018 (3)	0.0669 (11)	
O3	0.4465 (5)	0.56765 (18)	0.8725 (3)	0.0732 (13)	
O4	0.1747 (5)	0.3525 (2)	0.6905 (4)	0.0844 (14)	
O5	0.4055 (5)	0.7571 (2)	0.9173 (4)	0.1113 (18)	
O6	0.1856 (8)	0.7690 (3)	0.9745 (6)	0.150 (3)	
O7	0.2544 (6)	0.6820 (2)	0.9289 (5)	0.132 (2)	
O8	0.2123 (7)	0.7500 (3)	0.8194 (5)	0.136 (2)	
N1	0.3467 (5)	0.53815 (19)	0.6778 (3)	0.0568 (13)	
N2	0.4665 (5)	0.43982 (19)	0.7198 (3)	0.0499 (11)	
N3	0.2912 (5)	0.5010 (2)	0.8922 (3)	0.0568 (12)	
N4	0.1743 (5)	0.4105 (2)	0.8140 (4)	0.0594 (13)	
C1	0.2907 (7)	0.5872 (3)	0.6577 (5)	0.0703 (18)	
C2	0.3467 (9)	0.6270 (3)	0.5968 (5)	0.080 (2)	
H2	0.3015	0.6604	0.5832	0.096*	
C3	0.4689 (8)	0.6151 (3)	0.5585 (5)	0.077 (2)	
H3	0.5072	0.6409	0.5175	0.092*	
C4	0.5403 (7)	0.5652 (3)	0.5781 (4)	0.0682 (18)	
C5	0.6692 (7)	0.5514 (3)	0.5426 (5)	0.0719 (18)	
H5	0.7121	0.5758	0.5013	0.086*	
C6	0.7305 (7)	0.5035 (3)	0.5679 (5)	0.0755 (19)	
H6	0.8171	0.4959	0.5451	0.091*	
C7	0.6676 (6)	0.4634 (3)	0.6290 (4)	0.0591 (16)	
C8	0.7256 (6)	0.4127 (3)	0.6569 (5)	0.0645 (17)	
H8	0.8123	0.4032	0.6360	0.077*	
C9	0.6588 (7)	0.3770 (3)	0.7134 (5)	0.0641 (17)	
H9	0.6984	0.3432	0.7319	0.077*	
C10	0.5271 (6)	0.3923 (2)	0.7438 (4)	0.0558 (15)	
C11	0.5375 (6)	0.4756 (2)	0.6638 (4)	0.0502 (14)	
C12	0.4721 (6)	0.5278 (2)	0.6399 (4)	0.0559 (15)	
C13	0.3622 (7)	0.5429 (3)	0.9315 (5)	0.0649 (17)	
C14	0.3474 (8)	0.5577 (3)	1.0286 (5)	0.081 (2)	
H14	0.3994	0.5866	1.0555	0.098*	
C15	0.2577 (9)	0.5296 (3)	1.0820 (5)	0.085 (2)	
H15	0.2472	0.5400	1.1455	0.102*	
C16	0.1785 (8)	0.4845 (3)	1.0434 (5)	0.0703 (19)	
C17	0.0836 (9)	0.4528 (4)	1.0953 (5)	0.085 (2)	
H17	0.0657	0.4623	1.1582	0.102*	
C18	0.0187 (8)	0.4085 (3)	1.0533 (5)	0.084 (2)	
H18	−0.0455	0.3887	1.0876	0.101*	
C19	0.0465 (7)	0.3918 (3)	0.9581 (5)	0.0691 (18)	
C20	−0.0109 (7)	0.3445 (3)	0.9093 (6)	0.081 (2)	
H20	−0.0748	0.3225	0.9400	0.097*	
C21	0.0243 (7)	0.3310 (3)	0.8216 (6)	0.079 (2)	
H21	−0.0151	0.3003	0.7908	0.095*	
C22	0.1240 (7)	0.3645 (3)	0.7745 (5)	0.0692 (18)	
C23	0.1387 (6)	0.4240 (2)	0.9053 (4)	0.0586 (15)	
C24	0.2031 (6)	0.4713 (2)	0.9485 (4)	0.0558 (15)	
C25	0.0873 (13)	0.6395 (5)	0.6618 (10)	0.165 (4)	
H25A	0.1307	0.6751	0.6756	0.198*	
H25B	0.0838	0.6348	0.5928	0.198*	
C26	−0.0473 (14)	0.6412 (6)	0.6939 (11)	0.204 (6)	
H26A	−0.1004	0.6121	0.6633	0.306*	
H26B	−0.0875	0.6768	0.6783	0.306*	
H26C	−0.0463	0.6359	0.7619	0.306*	
C27	0.5010 (8)	0.3041 (3)	0.8233 (5)	0.081 (2)	
H27A	0.5860	0.3055	0.8613	0.097*	
H27B	0.5172	0.2838	0.7648	0.097*	
C28	0.3898 (8)	0.2763 (3)	0.8787 (6)	0.102 (3)	
H28A	0.3661	0.2996	0.9316	0.153*	
H28B	0.4223	0.2409	0.9023	0.153*	
H28C	0.3103	0.2707	0.8375	0.153*	
C29	0.5355 (7)	0.6122 (3)	0.9063 (5)	0.084 (2)	
H29A	0.5997	0.5984	0.9553	0.101*	
H29B	0.4818	0.6420	0.9337	0.101*	
C30	0.6118 (8)	0.6336 (3)	0.8219 (6)	0.105 (3)	
H30A	0.6665	0.6041	0.7965	0.158*	
H30B	0.6704	0.6639	0.8419	0.158*	
H30C	0.5473	0.6464	0.7734	0.158*	
C31	0.1123 (10)	0.3080 (4)	0.6354 (7)	0.129 (3)	
H31A	0.0858	0.2781	0.6778	0.155*	
H31B	0.0303	0.3217	0.6020	0.155*	
C32	0.2069 (11)	0.2869 (5)	0.5671 (8)	0.159 (4)	
H32A	0.1957	0.3073	0.5082	0.238*	
H32B	0.1887	0.2481	0.5555	0.238*	
H32C	0.2992	0.2914	0.5919	0.238*	

### Atomic displacement parameters (Å^2^)

**Table:**

	*U*^11^	*U*^22^	*U*^33^	*U*^12^	*U*^13^	*U*^23^
Cu1	0.0593 (5)	0.0552 (5)	0.0568 (5)	−0.0024 (4)	−0.0015 (4)	−0.0043 (3)
Cl1	0.0666 (11)	0.1277 (18)	0.1029 (15)	−0.0222 (11)	−0.0256 (10)	0.0300 (12)
Cl2	0.0762 (12)	0.0558 (10)	0.0921 (13)	0.0037 (8)	0.0009 (10)	0.0131 (9)
O1	0.094 (4)	0.070 (3)	0.103 (4)	0.026 (3)	−0.001 (3)	0.008 (3)
O2	0.076 (3)	0.048 (2)	0.077 (3)	0.004 (2)	0.000 (2)	0.003 (2)
O3	0.084 (3)	0.058 (3)	0.076 (3)	−0.012 (2)	−0.013 (3)	−0.015 (2)
O4	0.073 (3)	0.087 (3)	0.095 (4)	−0.027 (3)	0.014 (3)	−0.039 (3)
O5	0.076 (3)	0.120 (4)	0.137 (4)	−0.012 (3)	−0.012 (3)	0.026 (3)
O6	0.133 (5)	0.141 (5)	0.179 (6)	0.010 (4)	0.025 (4)	−0.063 (5)
O7	0.122 (4)	0.073 (3)	0.201 (6)	0.006 (3)	0.039 (4)	0.038 (4)
O8	0.148 (5)	0.130 (5)	0.126 (5)	−0.034 (4)	−0.043 (4)	0.018 (4)
N1	0.058 (3)	0.055 (3)	0.057 (3)	0.005 (2)	−0.010 (3)	−0.006 (2)
N2	0.055 (3)	0.047 (3)	0.047 (3)	−0.004 (2)	−0.006 (2)	−0.005 (2)
N3	0.064 (3)	0.048 (3)	0.058 (3)	0.009 (3)	−0.010 (3)	−0.008 (2)
N4	0.056 (3)	0.059 (3)	0.063 (3)	−0.003 (2)	0.001 (2)	−0.010 (3)
C1	0.068 (5)	0.068 (5)	0.073 (5)	0.006 (4)	−0.014 (4)	−0.006 (4)
C2	0.090 (6)	0.060 (4)	0.088 (5)	0.000 (4)	−0.020 (4)	0.015 (4)
C3	0.096 (6)	0.054 (4)	0.078 (5)	−0.018 (4)	−0.023 (4)	0.017 (3)
C4	0.078 (5)	0.067 (4)	0.059 (4)	−0.023 (4)	−0.021 (4)	−0.002 (3)
C5	0.062 (4)	0.081 (5)	0.073 (5)	−0.021 (4)	−0.004 (4)	0.010 (4)
C6	0.050 (4)	0.106 (6)	0.070 (5)	−0.017 (4)	0.000 (3)	−0.003 (4)
C7	0.052 (4)	0.076 (4)	0.048 (3)	−0.011 (3)	−0.009 (3)	−0.012 (3)
C8	0.052 (4)	0.075 (5)	0.066 (4)	0.009 (3)	−0.001 (3)	−0.021 (4)
C9	0.062 (4)	0.063 (4)	0.068 (4)	0.009 (3)	−0.005 (3)	−0.009 (3)
C10	0.067 (4)	0.048 (4)	0.051 (4)	0.000 (3)	−0.007 (3)	−0.010 (3)
C11	0.052 (3)	0.055 (3)	0.043 (3)	−0.001 (3)	−0.011 (3)	−0.005 (3)
C12	0.061 (4)	0.054 (4)	0.051 (3)	−0.014 (3)	−0.015 (3)	0.000 (3)
C13	0.073 (4)	0.056 (4)	0.064 (4)	0.014 (3)	−0.018 (4)	−0.010 (3)
C14	0.112 (6)	0.063 (5)	0.066 (5)	0.005 (4)	−0.028 (4)	−0.015 (4)
C15	0.129 (7)	0.080 (5)	0.046 (4)	0.028 (5)	−0.010 (4)	−0.009 (4)
C16	0.093 (5)	0.068 (4)	0.049 (4)	0.025 (4)	−0.009 (4)	−0.001 (3)
C17	0.104 (6)	0.099 (6)	0.051 (4)	0.022 (5)	0.009 (4)	0.009 (4)
C18	0.087 (6)	0.097 (6)	0.071 (5)	0.022 (5)	0.015 (4)	0.024 (4)
C19	0.062 (4)	0.077 (5)	0.068 (5)	0.012 (4)	0.007 (3)	0.015 (4)
C20	0.063 (4)	0.078 (5)	0.102 (6)	−0.012 (4)	0.012 (4)	0.015 (4)
C21	0.067 (5)	0.080 (5)	0.091 (6)	−0.020 (4)	0.009 (4)	−0.008 (4)
C22	0.063 (4)	0.070 (4)	0.075 (5)	−0.009 (3)	0.005 (4)	−0.018 (4)
C23	0.066 (4)	0.059 (4)	0.050 (4)	0.005 (3)	0.001 (3)	0.001 (3)
C24	0.069 (4)	0.047 (3)	0.051 (4)	0.011 (3)	−0.003 (3)	0.001 (3)
C25	0.155 (8)	0.153 (8)	0.189 (8)	0.054 (7)	0.043 (7)	0.040 (7)
C26	0.191 (9)	0.211 (10)	0.209 (9)	0.024 (8)	−0.015 (8)	0.039 (8)
C27	0.100 (5)	0.060 (4)	0.084 (5)	0.007 (4)	0.000 (4)	0.004 (3)
C28	0.117 (6)	0.076 (5)	0.113 (6)	−0.001 (4)	−0.004 (5)	0.024 (4)
C29	0.082 (5)	0.066 (4)	0.103 (5)	0.006 (4)	−0.033 (4)	−0.020 (4)
C30	0.098 (5)	0.097 (5)	0.120 (6)	−0.035 (5)	−0.007 (5)	−0.008 (5)
C31	0.117 (6)	0.147 (7)	0.124 (6)	−0.049 (6)	0.009 (5)	−0.066 (6)
C32	0.178 (8)	0.152 (7)	0.145 (7)	0.004 (7)	−0.006 (7)	−0.066 (6)

### Geometric parameters (Å, °)

**Table:**

Cu1—N1	1.999 (5)	C13—C14	1.414 (9)
Cu1—N4	2.003 (5)	C14—C15	1.347 (10)
Cu1—N2	2.158 (5)	C14—H14	0.9300
Cu1—N3	2.162 (5)	C15—C16	1.425 (10)
Cu1—Cl1	2.2847 (19)	C15—H15	0.9300
Cl2—O6	1.392 (6)	C16—C24	1.392 (9)
Cl2—O7	1.404 (5)	C16—C17	1.413 (10)
Cl2—O8	1.408 (6)	C17—C18	1.359 (10)
Cl2—O5	1.411 (5)	C17—H17	0.9300
O1—C1	1.349 (8)	C18—C19	1.424 (9)
O1—C25	1.447 (11)	C18—H18	0.9300
O2—C10	1.353 (7)	C19—C23	1.411 (9)
O2—C27	1.441 (7)	C19—C20	1.427 (10)
O3—C13	1.324 (8)	C20—C21	1.324 (9)
O3—C29	1.446 (7)	C20—H20	0.9300
O4—C22	1.320 (7)	C21—C22	1.434 (9)
O4—C31	1.438 (8)	C21—H21	0.9300
N1—C1	1.322 (8)	C23—C24	1.420 (8)
N1—C12	1.370 (8)	C25—C26	1.401 (9)
N2—C10	1.321 (7)	C25—H25A	0.9700
N2—C11	1.363 (7)	C25—H25B	0.9700
N3—C13	1.328 (8)	C26—H26A	0.9600
N3—C24	1.380 (7)	C26—H26B	0.9600
N4—C22	1.320 (7)	C26—H26C	0.9600
N4—C23	1.371 (7)	C27—C28	1.508 (7)
C1—C2	1.400 (9)	C27—H27A	0.9700
C2—C3	1.352 (10)	C27—H27B	0.9700
C2—H2	0.9300	C28—H28A	0.9600
C3—C4	1.406 (9)	C28—H28B	0.9600
C3—H3	0.9300	C28—H28C	0.9600
C4—C5	1.405 (9)	C29—C30	1.505 (8)
C4—C12	1.422 (8)	C29—H29A	0.9700
C5—C6	1.336 (10)	C29—H29B	0.9700
C5—H5	0.9300	C30—H30A	0.9600
C6—C7	1.436 (9)	C30—H30B	0.9600
C6—H6	0.9300	C30—H30C	0.9600
C7—C8	1.390 (8)	C31—C32	1.439 (8)
C7—C11	1.403 (8)	C31—H31A	0.9700
C8—C9	1.345 (9)	C31—H31B	0.9700
C8—H8	0.9300	C32—H32A	0.9600
C9—C10	1.414 (8)	C32—H32B	0.9600
C9—H9	0.9300	C32—H32C	0.9600
C11—C12	1.439 (8)		
			
N1—Cu1—N4	176.8 (2)	C16—C15—H15	119.2
N1—Cu1—N2	80.38 (19)	C24—C16—C17	120.2 (7)
N4—Cu1—N2	102.79 (19)	C24—C16—C15	115.2 (7)
N1—Cu1—N3	100.03 (19)	C17—C16—C15	124.6 (7)
N4—Cu1—N3	79.6 (2)	C18—C17—C16	120.0 (7)
N2—Cu1—N3	101.22 (17)	C18—C17—H17	120.0
N1—Cu1—Cl1	91.64 (15)	C16—C17—H17	120.0
N4—Cu1—Cl1	86.22 (15)	C17—C18—C19	121.6 (7)
N2—Cu1—Cl1	130.20 (13)	C17—C18—H18	119.2
N3—Cu1—Cl1	128.54 (15)	C19—C18—H18	119.2
O6—Cl2—O7	109.7 (4)	C23—C19—C18	118.5 (7)
O6—Cl2—O8	106.2 (5)	C23—C19—C20	115.5 (6)
O7—Cl2—O8	107.2 (4)	C18—C19—C20	125.9 (7)
O6—Cl2—O5	112.4 (4)	C21—C20—C19	121.8 (7)
O7—Cl2—O5	111.9 (4)	C21—C20—H20	119.1
O8—Cl2—O5	109.3 (4)	C19—C20—H20	119.1
C1—O1—C25	114.1 (7)	C20—C21—C22	119.2 (7)
C10—O2—C27	118.6 (5)	C20—C21—H21	120.4
C13—O3—C29	120.3 (5)	C22—C21—H21	120.4
C22—O4—C31	118.3 (6)	O4—C22—N4	114.1 (6)
C1—N1—C12	116.5 (6)	O4—C22—C21	124.4 (6)
C1—N1—Cu1	128.7 (5)	N4—C22—C21	121.5 (7)
C12—N1—Cu1	114.5 (4)	N4—C23—C19	122.8 (6)
C10—N2—C11	117.2 (5)	N4—C23—C24	117.5 (6)
C10—N2—Cu1	132.6 (4)	C19—C23—C24	119.6 (6)
C11—N2—Cu1	110.1 (4)	N3—C24—C16	123.7 (6)
C13—N3—C24	118.7 (5)	N3—C24—C23	116.3 (5)
C13—N3—Cu1	131.9 (5)	C16—C24—C23	119.9 (6)
C24—N3—Cu1	109.0 (4)	C26—C25—O1	115.2 (11)
C22—N4—C23	119.0 (6)	C26—C25—H25A	108.5
C22—N4—Cu1	126.2 (5)	O1—C25—H25A	108.5
C23—N4—Cu1	113.3 (4)	C26—C25—H25B	108.5
N1—C1—O1	112.8 (6)	O1—C25—H25B	108.5
N1—C1—C2	124.6 (7)	H25A—C25—H25B	107.5
O1—C1—C2	122.7 (7)	C25—C26—H26A	109.5
C3—C2—C1	117.6 (7)	C25—C26—H26B	109.5
C3—C2—H2	121.2	H26A—C26—H26B	109.5
C1—C2—H2	121.2	C25—C26—H26C	109.5
C2—C3—C4	122.6 (7)	H26A—C26—H26C	109.5
C2—C3—H3	118.7	H26B—C26—H26C	109.5
C4—C3—H3	118.7	O2—C27—C28	106.2 (6)
C5—C4—C3	125.0 (7)	O2—C27—H27A	110.5
C5—C4—C12	120.5 (6)	C28—C27—H27A	110.5
C3—C4—C12	114.5 (7)	O2—C27—H27B	110.5
C6—C5—C4	120.4 (7)	C28—C27—H27B	110.5
C6—C5—H5	119.8	H27A—C27—H27B	108.7
C4—C5—H5	119.8	C27—C28—H28A	109.5
C5—C6—C7	122.4 (7)	C27—C28—H28B	109.5
C5—C6—H6	118.8	H28A—C28—H28B	109.5
C7—C6—H6	118.8	C27—C28—H28C	109.5
C8—C7—C11	116.7 (6)	H28A—C28—H28C	109.5
C8—C7—C6	125.0 (6)	H28B—C28—H28C	109.5
C11—C7—C6	118.4 (6)	O3—C29—C30	107.5 (6)
C9—C8—C7	121.3 (6)	O3—C29—H29A	110.2
C9—C8—H8	119.4	C30—C29—H29A	110.2
C7—C8—H8	119.4	O3—C29—H29B	110.2
C8—C9—C10	118.3 (6)	C30—C29—H29B	110.2
C8—C9—H9	120.9	H29A—C29—H29B	108.5
C10—C9—H9	120.9	C29—C30—H30A	109.5
N2—C10—O2	113.9 (5)	C29—C30—H30B	109.5
N2—C10—C9	123.3 (6)	H30A—C30—H30B	109.5
O2—C10—C9	122.8 (6)	C29—C30—H30C	109.5
N2—C11—C7	123.3 (5)	H30A—C30—H30C	109.5
N2—C11—C12	116.8 (5)	H30B—C30—H30C	109.5
C7—C11—C12	119.9 (6)	C32—C31—O4	110.3 (7)
N1—C12—C4	124.2 (6)	C32—C31—H31A	109.6
N1—C12—C11	117.4 (5)	O4—C31—H31A	109.6
C4—C12—C11	118.4 (6)	C32—C31—H31B	109.6
O3—C13—N3	114.0 (6)	O4—C31—H31B	109.6
O3—C13—C14	124.8 (6)	H31A—C31—H31B	108.1
N3—C13—C14	121.2 (7)	C31—C32—H32A	109.5
C15—C14—C13	119.6 (7)	C31—C32—H32B	109.5
C15—C14—H14	120.2	H32A—C32—H32B	109.5
C13—C14—H14	120.2	C31—C32—H32C	109.5
C14—C15—C16	121.5 (7)	H32A—C32—H32C	109.5
C14—C15—H15	119.2	H32B—C32—H32C	109.5
			
N2—Cu1—N1—C1	−178.8 (5)	C1—N1—C12—C4	−2.9 (8)
N3—Cu1—N1—C1	−79.0 (5)	Cu1—N1—C12—C4	171.3 (4)
Cl1—Cu1—N1—C1	50.7 (5)	C1—N1—C12—C11	177.7 (5)
N2—Cu1—N1—C12	7.8 (4)	Cu1—N1—C12—C11	−8.1 (6)
N3—Cu1—N1—C12	107.7 (4)	C5—C4—C12—N1	179.8 (5)
Cl1—Cu1—N1—C12	−122.7 (4)	C3—C4—C12—N1	0.4 (8)
N1—Cu1—N2—C10	176.9 (5)	C5—C4—C12—C11	−0.8 (8)
N4—Cu1—N2—C10	−3.3 (5)	C3—C4—C12—C11	179.9 (5)
N3—Cu1—N2—C10	78.4 (5)	N2—C11—C12—N1	2.2 (7)
Cl1—Cu1—N2—C10	−99.2 (5)	C7—C11—C12—N1	−178.4 (5)
N1—Cu1—N2—C11	−6.5 (3)	N2—C11—C12—C4	−177.2 (5)
N4—Cu1—N2—C11	173.4 (3)	C7—C11—C12—C4	2.1 (7)
N3—Cu1—N2—C11	−104.9 (3)	C29—O3—C13—N3	−177.0 (5)
Cl1—Cu1—N2—C11	77.5 (4)	C29—O3—C13—C14	2.0 (9)
N1—Cu1—N3—C13	−11.7 (5)	C24—N3—C13—O3	178.2 (5)
N4—Cu1—N3—C13	171.5 (5)	Cu1—N3—C13—O3	−10.2 (8)
N2—Cu1—N3—C13	70.4 (5)	C24—N3—C13—C14	−0.8 (8)
Cl1—Cu1—N3—C13	−111.9 (5)	Cu1—N3—C13—C14	170.8 (4)
N1—Cu1—N3—C24	160.6 (4)	O3—C13—C14—C15	179.7 (6)
N4—Cu1—N3—C24	−16.2 (4)	N3—C13—C14—C15	−1.4 (10)
N2—Cu1—N3—C24	−117.4 (4)	C13—C14—C15—C16	1.2 (11)
Cl1—Cu1—N3—C24	60.3 (4)	C14—C15—C16—C24	1.2 (10)
N2—Cu1—N4—C22	−76.5 (5)	C14—C15—C16—C17	179.3 (7)
N3—Cu1—N4—C22	−175.8 (5)	C24—C16—C17—C18	1.6 (10)
Cl1—Cu1—N4—C22	53.9 (5)	C15—C16—C17—C18	−176.4 (7)
N2—Cu1—N4—C23	117.5 (4)	C16—C17—C18—C19	1.9 (11)
N3—Cu1—N4—C23	18.2 (4)	C17—C18—C19—C23	−3.2 (10)
Cl1—Cu1—N4—C23	−112.1 (4)	C17—C18—C19—C20	176.8 (7)
C12—N1—C1—O1	−175.3 (5)	C23—C19—C20—C21	2.2 (10)
Cu1—N1—C1—O1	11.5 (8)	C18—C19—C20—C21	−177.8 (7)
C12—N1—C1—C2	3.9 (9)	C19—C20—C21—C22	0.7 (11)
Cu1—N1—C1—C2	−169.4 (5)	C31—O4—C22—N4	−171.0 (7)
C25—O1—C1—N1	−161.5 (8)	C31—O4—C22—C21	9.8 (11)
C25—O1—C1—C2	19.3 (11)	C23—N4—C22—O4	−173.8 (5)
N1—C1—C2—C3	−2.3 (10)	Cu1—N4—C22—O4	21.0 (8)
O1—C1—C2—C3	176.8 (6)	C23—N4—C22—C21	5.4 (9)
C1—C2—C3—C4	−0.5 (10)	Cu1—N4—C22—C21	−159.8 (5)
C2—C3—C4—C5	−178.0 (7)	C20—C21—C22—O4	174.4 (7)
C2—C3—C4—C12	1.3 (9)	C20—C21—C22—N4	−4.8 (11)
C3—C4—C5—C6	178.0 (6)	C22—N4—C23—C19	−2.3 (9)
C12—C4—C5—C6	−1.3 (9)	Cu1—N4—C23—C19	164.8 (5)
C4—C5—C6—C7	2.0 (10)	C22—N4—C23—C24	175.1 (5)
C5—C6—C7—C8	179.2 (6)	Cu1—N4—C23—C24	−17.8 (6)
C5—C6—C7—C11	−0.6 (9)	C18—C19—C23—N4	178.5 (6)
C11—C7—C8—C9	0.8 (8)	C20—C19—C23—N4	−1.5 (9)
C6—C7—C8—C9	−179.1 (6)	C18—C19—C23—C24	1.2 (9)
C7—C8—C9—C10	0.2 (9)	C20—C19—C23—C24	−178.8 (6)
C11—N2—C10—O2	178.0 (4)	C13—N3—C24—C16	3.5 (8)
Cu1—N2—C10—O2	−5.6 (7)	Cu1—N3—C24—C16	−170.0 (5)
C11—N2—C10—C9	−0.9 (8)	C13—N3—C24—C23	−174.7 (5)
Cu1—N2—C10—C9	175.5 (4)	Cu1—N3—C24—C23	11.8 (6)
C27—O2—C10—N2	172.7 (5)	C17—C16—C24—N3	178.2 (6)
C27—O2—C10—C9	−8.4 (8)	C15—C16—C24—N3	−3.6 (9)
C8—C9—C10—N2	−0.2 (9)	C17—C16—C24—C23	−3.7 (9)
C8—C9—C10—O2	−179.0 (5)	C15—C16—C24—C23	174.5 (6)
C10—N2—C11—C7	2.1 (7)	N4—C23—C24—N3	3.0 (8)
Cu1—N2—C11—C7	−175.1 (4)	C19—C23—C24—N3	−179.5 (5)
C10—N2—C11—C12	−178.6 (5)	N4—C23—C24—C16	−175.3 (5)
Cu1—N2—C11—C12	4.2 (5)	C19—C23—C24—C16	2.2 (8)
C8—C7—C11—N2	−2.1 (8)	C1—O1—C25—C26	166.7 (12)
C6—C7—C11—N2	177.8 (5)	C10—O2—C27—C28	−173.4 (5)
C8—C7—C11—C12	178.6 (5)	C13—O3—C29—C30	−176.9 (6)
C6—C7—C11—C12	−1.5 (8)	C22—O4—C31—C32	−158.3 (8)
